# Targeted deletion of floral development genes in *Arabidopsis* with CRISPR/Cas9 using the RNA endoribonuclease Csy4 processing system

**DOI:** 10.1038/s41438-019-0179-6

**Published:** 2019-08-21

**Authors:** Yingzhu Liu, Yike Gao, Yaohui Gao, Qixiang Zhang

**Affiliations:** Beijing Key Laboratory of Ornamental Plants Germplasm Innovation and Molecular Breeding, National Engineering Research Center for Floriculture, College of Ornamental Horticulture and Landscape Architecture, Beijing Forestry University, 100083 Beijing, China

**Keywords:** Plant molecular biology, Molecular engineering in plants, Molecular engineering in plants

## Abstract

The formation of flowers in higher plants is controlled by complex gene regulatory networks. The study of floral development in *Arabidopsis* is promoted and maintained by transposon-tagged mutant lines. In this study, we report a CRISPR/Cas9 genome-editing system based on RNA endoribonuclease Csy4 processing to induce high-efficiency and inheritable targeted deletion of transcription factors involved in floral development in *Arabidopsis*. Using *AP1*, *SVP*, and *TFL1* as the target genes, multisite and multiple-gene mutations were achieved with a tandemly arrayed Csy4-sgRNA architecture to express multiplexed sgRNAs from a single transcript driven by the Pol II promoter in transgenic lines. Targeted deletions of chromosomal fragments between the first exon and second exon in either one or three genes were generated by using a single binary vector. Interestingly, the efficiency of site-targeted deletion was comparable to that of indel mutation with the multiplexed sgRNAs. DNA sequencing analysis of RT-PCR products showed that targeted deletions of *AP1* and *TFL1* could lead to frameshift mutations and introduce premature stop codons to disrupt the open-reading frames of the target genes. In addition, no RT-PCR amplified product was acquired after *SVP*-targeted deletion. Furthermore, the targeted deletions resulted in abnormal floral development in the mutant lines compared to that of wild-type plants. *AP1* and *SVP* mutations increased plant branching significantly, while *TFL1* mutant plants displayed a change from indeterminate to determinate inflorescences. Thus, our results demonstrate that CRISPR/Cas9 with the RNA endoribonuclease Csy4 processing system is an efficient tool to study floral development and improve floral traits rapidly and simply.

## Introduction

The CRISPR/Cas9 (clustered, regularly interspersed palindromic repeats-associated endonuclease 9) system has recently been developed into a powerful genome-editing technology with a role in revealing plant gene function and improving crops^1–5^. CRISPR/Cas9 with a single guide RNA (sgRNA) targeted to specific DNA sequences generates a DNA double-strand break (DSB) at a position three base pairs upstream of the protospacer adjacent motif (PAM) sequence. The resulting DSBs are repaired primarily by either nonhomologous end joining (NHEJ) or homology-directed repair (HDR) in vivo^6–8^. NHEJ occurs most frequently and can introduce indel mutations, such as random nucleotide deletions or insertions at the break site that induce a frameshift mutation in the target gene^6^. The targeted mutagenesis derived from genome editing by CRISPR/Cas9 is valuable for understanding the functions of specific genes, and it is becoming one of the most powerful tools to knock out an individual gene, as well as to target the insertion of a specific gene and/or control gene transcription^9–13^. However, the traditional CRISPR/Cas9 system is dependent on the RNA polymerase III (Pol III) promoter driving one sgRNA to generate one DSB at a target site in the genome^6,8,14–17^.

Previous studies have shown that mutations introduced by CRISPR/Cas9 using one sgRNA are difficult to identify by PCR analysis, and techniques are limited to homology analysis by DNA sequencing^9,12,18–21^. It was also found that the mutant exons generated by CRISPR/Cas9 with one sgRNA can lead to exon skipping^19^. Multiplex gene knockout has been performed by CRISPR/Cas9 using a single sgRNA targeting the conserved regions of multiple genes in a family^22^ or using multiplexed sgRNAs that were expressed from a single transcript and could generate individual functional sgRNAs after processing by exogenous ribozymes^23^, Csy4^24,25^, or the plant endogenous transfer RNA (tRNA)-processing system^26^. Based on these technologies, the CRISPR/Cas9 toolkits enable genome editing to achieve deletion of chromosomal fragments in a target site with a pair of sgRNAs.

Flowering is an important developmental phase switch in the life cycle of higher plants. In flowering plants, *APETALA1* (*AP1*), *SHORT VEGETATIVE PHASE* (*SVP*), and *TERMINAL FLOWER1* (*TFL1*) are essential network genes that control the transition from a vegetative state to a reproductive state and the formation of flowers^27,28^. *AP1* is a MADS-box transcriptional regulator that initiates flower development, which is one of the key regulatory steps of plant flowering activated by flowering locus T^27,29^. Meanwhile, *SVP* is a key conserved flowering repressor that determines inflorescence architecture by acting to suppress *TFL1* in the emerging floral meristem both redundantly and directly, and causes high levels of inflorescence branching in flowering plants^30^. *TFL1* acts as a repressor of floral initiation, regulates indeterminate conversion to a determinate architecture, controls inflorescence development and maintains the inflorescence meristem in a vegetative state via the repression of *AP1* invasion of the central part of the shoot apex^31^.

In this study, we arrayed sgRNA sequences with a 20 bp Csy4 hairpin sequence and produced multiplexed sgRNAs processed by the RNA endoribonuclease Csy4 from *Pseudomonas aeruginosa* in combination with CRISPR/Cas9 to knock out transcription factors involved in floral development in *Arabidopsis*. *AP1*, *SVP*, and *TFL1* were selected as the target genes to produce targeted deletions of chromosomal fragments between two DSBs separately or simultaneously. We showed that CRISPR/Cas9 using the RNA endoribonuclease Csy4-processing system successfully knocked out the target genes and generated mutant lines rapidly and simply. In addition, the frameshift mutations caused by the targeted deletions could be stably propagated. Our results demonstrate that the CRISPR/Cas9 system with RNA endoribonuclease Csy4 processing is a powerful tool for studying floral development with a variety of genes.

## Results

### Target site selection for *AP1*, *SVP*, and *TFL1*

To design the protospacer at the target site of *AP1*, *SVP*, and *TFL1*, we determined the genomic sequences of these genes through PCR using gene-specific primer pairs (AP-1/AP-2, SVP-1/SVP-2, and TFL-1/TFL-2 for *AP1*, *SVP*, and *TFL1*, respectively) and analysis of the nucleotide sequences by molecular cloning and DNA sequencing. The corresponding primers are provided in Table S1. The DNA sequences were compared to the corresponding loci in the TAIR database (https://www.arabidopsis.org/) as listed below: *AP1* for AT1G69120.1, located on chromosome 1; *SVP* for AT2G22540, located on chromosome 2; and *TFL1* for AT5G03840, located on chromosome 5. Finally, target sites were selected in the first and second exons of the *AP1*, *SVP*, and *TFL1* genomic sequences (Table 1).

**Table 1:** Summary of targeted deletions with the CRISPR/Cas9 system

# sgRNAs	Sequence 5′–3′	PAM 5′–3′	GC content (%)	Target loci	Deletion mutants/# of Hyg+T1 plants
sgRNA1	GGGGTAGGGTTCAATTGAAG	AGG	50	*AP1* exon 1	8/23
sgRNA2	AGATACTTGAACGCTATGAG	AGG	40	*AP1* exon 2	
sgRNA3	GACATCGGCGTCGCAGAGAA	CGG	60	*SVP* exon 1	5/17
sgRNA4	ACTGCAAGTTATGCCTCTCT	AGG	45	*SVP* exon 2	
sgRNA5	CTTCTGTTTCCTCCAAGCCT	AGG	50	*TFL1* exon 1	10/30
sgRNA6	ATGATAGACCCAGATGTTCC	AGG	45	*TFL1* exon 2	

### Assembly of CRISPR/Cas9 vectors

**Fig. 1:**
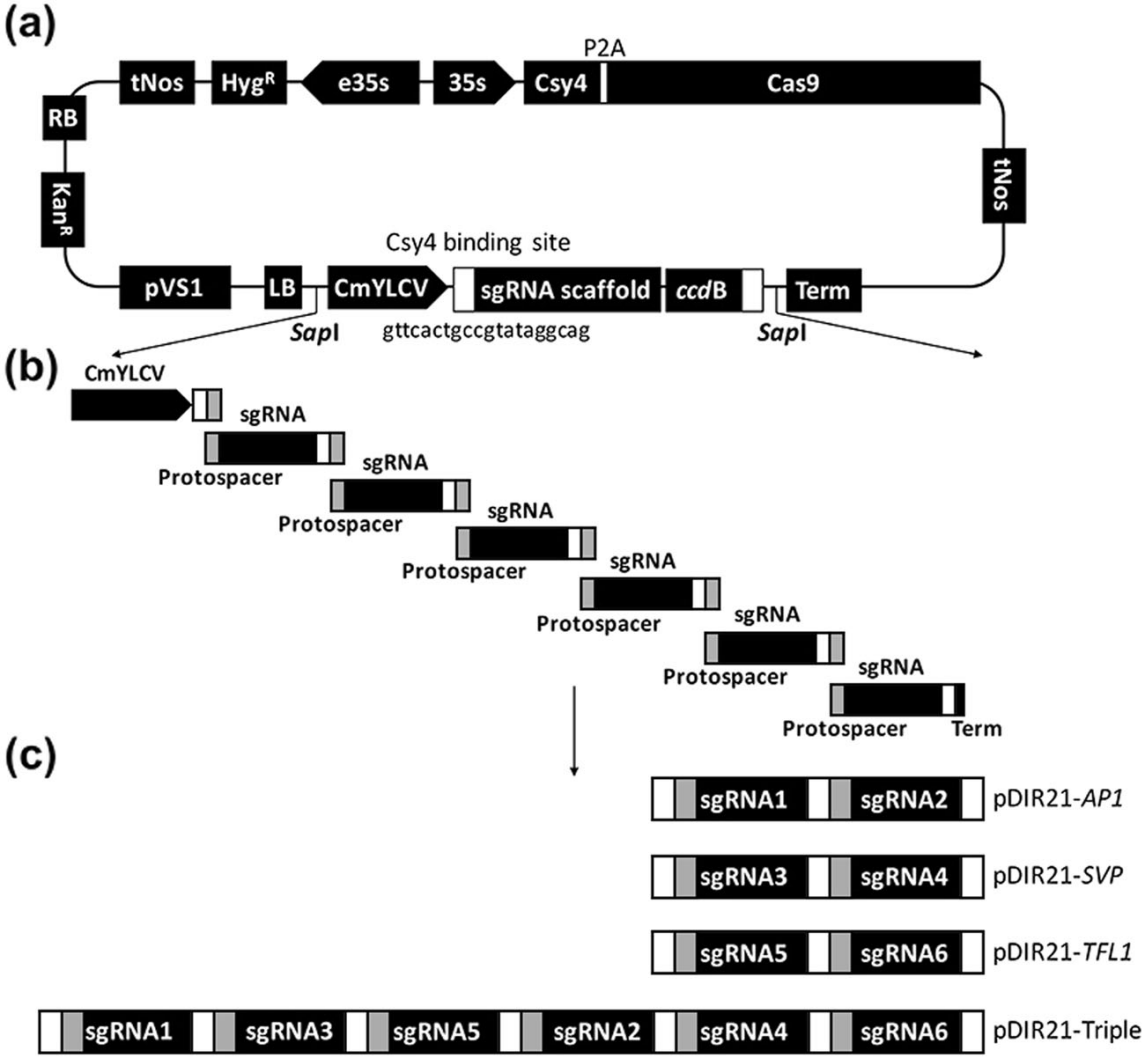
A schematic diagram of the CRISPR/Cas9 system with the multiple-sgRNA expression cassette. **a** The structure of the direct cloning binary vector pDIR21 was designed to speed up the cloning process of sgRNA and the transformation elements with CRISPR/Cas9. tNOS, CaMV poly(A) signal; Hyg hygromycin B phosphotransferase, e35s enhanced CaMV 35S promoter, 35s CaMV 35S promoter, Csy4 RNA endoribonuclease Csy4 from *Pseudomonas aeruginosa,* P2A self-splicing 2A Peptide derived from *Porcine teschovirus-1*, Cas9 Cas9 endonuclease, CmYLCV *Cestrum yellow leaf curling virus* promoter, sgRNA single guide RNA targeting specific sequence of genome locus, Csy4-binding site, a 20 bp sequence of Csy4 recognized hairpin structure, *ccd*B, lethal gene in *E. coli*. **b** Illustration of cloning of multiplexed sgRNA cassettes into the CRISPR/Cas9 binary vector by the Gibson assembly method. The 20nt protospacer sequences are overlapping regions for assembly, shown as gray boxes. **c** Overall structure of the multiplexed sgRNAs in intermediate vectors for targeted deletion of *AP1*, *SVP*, and *TFL1* in *Arabidopsis*

In an attempt to construct genome-editing vectors, we used the backbone of pDIRECT-21 (Fig. 1a), which contained *Arabidopsis* codon-optimized Cas9 driven by the 35s promoter (35s) and the *Cestrum yellow leaf curling virus* (CmYLCV) promoter for multiplexed sgRNA transcription. To facilitate positive/negative selection of transgenic plants, a selection marker, hygromycin B phosphotransferase, which confers tolerance to hygromycin, driven by an enhanced 35s promoter was used. The CmYLCV promoter produces a single transcript with sgRNA cassettes separated by the 20-bp Csy4 hairpins for multiplexed sgRNA release^24^. Fragments containing sgRNAs that targeted *AP1*, *SVP*, and *TFL1* separately or simultaneously were assembled into pDIRECT-21 by the Gibson assembly method (Fig. 1b and Table S1). The assembled CRISPR/Cas9 vectors with the Csy4-processing system for target gene deletions are shown in Fig. 1c, and the sequences of the multiple sgRNAs are shown in Supplementary Information 1.

### Effective multiplexed CRISPR/Cas9-mediated targeted deletion

**Fig. 2:**
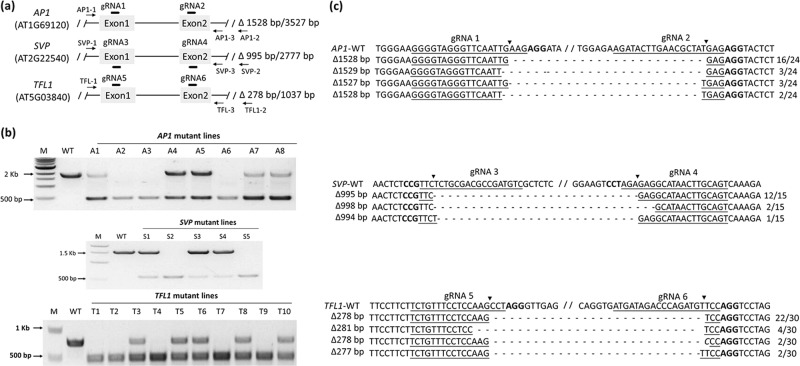
PCR detection revealed targeted deletions of *AP1*, *SVP*, and *TFL1* in T1 plants. **a** Map of three floral genes targeted for deletion using a pair of sgRNAs each. sgRNA sites are shown as black lines. Arrowheads represent the primers used to detect the deletions. **b** Lengths of PCR products from wild-type loci and the targeted deletions of *AP1*, *SVP*, and *TFL1*. **c** DNA sequences of representative deletion products. The sequence of the unmodified locus for each of the three genes is shown on the top. sgRNA target sites are underlined, and PAM sequences are in bold. Cleavage sites are shown as black arrowheads. Deleted sequences are shown with dots

To demonstrate the efficiency of the CRISPR/Cas9-generated deletions, primers were designed upstream and downstream of various target sites, including the loci edited by the vectors pDIR21-*AP1*, pDIR21-*SVP*, and pDIR21-*TFL1*. The amplified fragments covered the targeted deletions in each gene (Fig. 2a). The corresponding primers are provided in Table S1. We detected genome editing in these T1 plants by PCR amplification and sequenced three clones of each PCR product cloned into a T-vector. In the *AP1* lines, we obtained eight T1 lines that had the targeted deletion from 23 hygromycin-tolerant plants using a pair of AP-1/AP-3 primers (Fig. 2b). The analysis showed four types of genomic deletions at the target sites between gRNA1 and gRNA2 by DNA sequence analysis (Figs. 2c and S1). In *SVP* lines, five T1 lines that had the targeted deletion were obtained from 17 hygromycin-tolerant plants (Fig. 2b), and the analysis showed three types of genomic deletions at the target sites between gRNA3 and gRNA4 by DNA sequence analysis (Figs. 2c and S2). In *TFL1* lines, 10 T1 lines that had the targeted deletion were obtained from 30 hygromycin-tolerant plants (Fig. 2b), and the analysis showed four types of genomic deletions at the target sites between gRNA5 and gRNA6 according to DNA sequence analysis (Figs. 2c and S3).

We next tested the CRISPR/Cas9 genome-targeted deletion approach with the six multiplexed sgRNAs for *AP1*, *SVP*, and *TFL1* in pDIR21-Triple. From 48 hygromycin-tolerant plants, we obtained two lines with the three gene-targeted deletions; eight additional mutant lines were double mutants, and 15 had mutations in a single gene (Figs. S4–6). A summary of the targeted deletion efficiency by these four vectors in *Arabidopsis* T1 plants is shown in Table 1.

### Mutagenesis of target sites with multiplexed sgRNAs

We explored the effectiveness of the Csy4-processing system in mutagenesis of a single targeted site of *AP1*, *SVP*, and *TFL1* from pDIR21-Triple in 48 lines. We detected these T1 plants by PCR with the primers (Table S1) used to amplify the genomic regions containing the corresponding target sites. The PCR products from the six sites of the three genes were cloned and sequenced to investigate the mutation types. Overall, 109 valid sequences were obtained. A summary of the results is shown in Table 2.

**Table 2:** Targeted mutagenesis of a single target site with CRISPR/Cas9 using multiplexed sgRNAs

Target gene	Indel at sgRNA site #1 only	Indel at sgRNA site #2 only	Indel at both sgRNA sites	Inversion between sgRNA sites	Deletion between sgRNA sites	Total mutant plants
*AP1*	3	2	5	1	13	24
*SVP*	0	1	1	0	9	11
*TFL1*	2	1	15	2	15	35

**Fig. 3:**
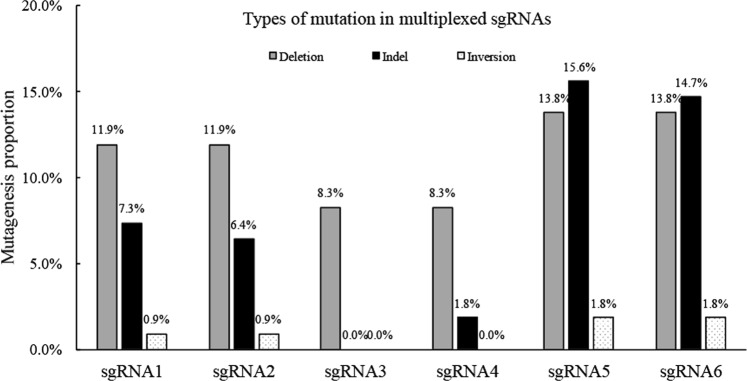
Genome-editing evaluation of six sgRNAs in the pDIR21-Triple vector. The editing efficiencies of different mutation types were determined by DNA sequencing in T1 plants

There are three types of mutagenesis: indel mutations, including insertion or deletion of nucleotides, deletions of a fragment between two sites and inversions of a fragment between two sites. The proportion of each type of mutation at the six target sites was calculated among the valid sequences, including single-target-site edits and deletions (Figs. 3 and S7–11). Thus, the results showed that the CRISPR/Cas9-processing system using the RNA endoribonuclease Csy4 provides efficient multiplexed, targeted gene deletion in *Arabidopsis* with multiplexed sgRNAs.

### Identification of homozygous targeted deletions in mutant lines

**Fig. 4:**
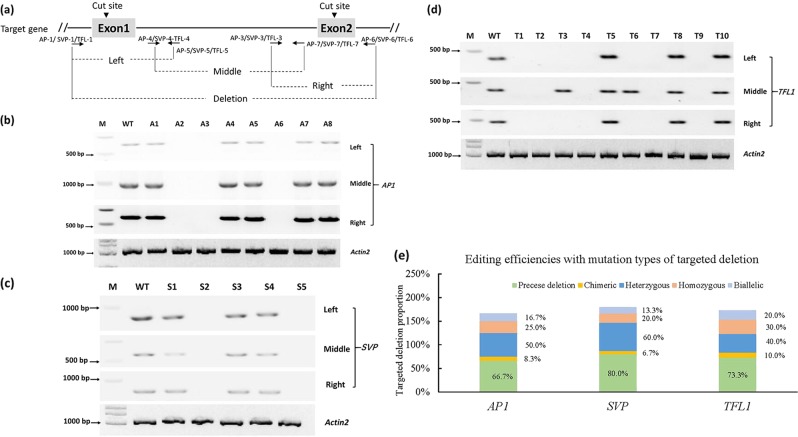
Verification of homozygous deletion mutant lines. **a** Primer sets were used to amplify the editing regions between two DSBs in *AP1*, *SVP*, and *TFL1* separately. **b**–**d** Agarose gel electrophoresis of the fragments of targeted genes. The absence of all three fragments indicated plants with homozygous deletions. WT wild type. **e** Efficiencies of targeted deletion with different mutation types in T1 plants

To further confirm the homozygous mutant status of the targeted genes in the selected plants, we investigated the mutations at each disrupted gene locus by using three pairs of primers covering the two cut sites and the regions between them (Fig. 4a). The corresponding primers are provided in Table S1. In the PCR detection results, the absence of all three fragments was indicative of homozygous deletions in *AP1* (Fig. 4b), *SVP* (Fig. 4c), and *TFL1* (Fig. 4d). The middle fragment for the T3 and T6 lines of the *TFL1* targeted deletion was amplified by the PCR primer pair TFL-4/TFL-7, showing that chromosomal translocations occurred between the two cut sites.

We analyzed the targeted deletion edit types in *Arabidopsis* T1 plants derived from the pDIR21-*AP1*, pDIR21-*SVP*, and pDIR21-*TFL1* constructs by DNA sequencing of three clones of each PCR fragment (Fig. 2b). Of the 207 obtained sequences, 16/24 (66.7%) of *AP1* mutation lines, 12/15 (80%) of SVP mutation lines, and 22/30 (73.3%) of TFL1 mutation lines showed precise deletions in each gene. Meanwhile, each targeted gene contained heterozygous, homozygous, chimeric and biallelic mutations at each site (Fig. 4e and Figs. S12,13). Finally, we identified precise, targeted, and homozygous deletions in A2 of *AP1*, S2 of *SVP* and T1 of *TFL1* lines.

### Targeted deletion for loss-of-function mutagenesis

**Fig. 5:**
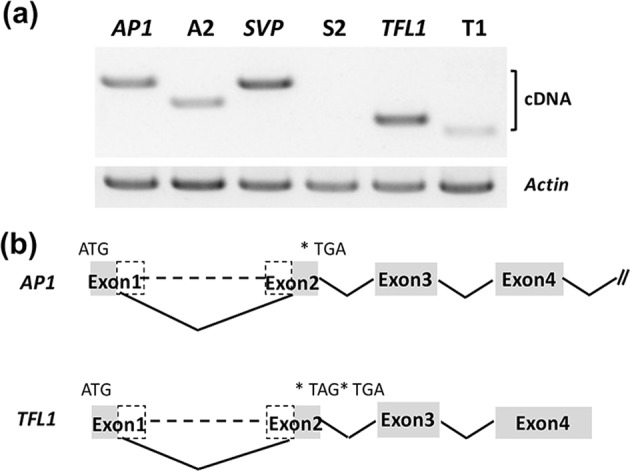
Targeted deletion led to frameshift mutations in *AP1* and *TFL1*. **a** Agarose gel electrophoresis of the targeted deletion gene mRNA transcripts from T1 events by RT-PCR. We used *Actin* as an internal control. **b** Schematic representation of the transcripts produced by *AP1* and *TFL1* deletion

To understand the impact of targeted deletion of the DNA fragments between exon 1 and exon 2 of *AP1*, *SVP*, and *TFL1* by CRISPR/Cas9 using two sgRNAs at the mRNA level in the homozygous mutation lines A2, S2, and T1, we screened the lines directly. We amplified the cDNA of the three genes by RT-PCR using oligo-d(T)_18_, which primed reverse transcription of mRNA from mutant T1 plants, and specific primer pairs binding the 5′ and 3′ UTR sequences of these genes were used separately. The corresponding primers are provided in Table S1. The results showed that the cDNA fragments of *AP1* and *TFL1* were amplified, but amplification failed for *SVP* (Fig. 5a). In the Sanger sequencing results, we identified the transcripts that corresponded to the known transcripts with partial sequences of exon 1 and exon 2 of *AP1* and *TFL1* separately (Figs. 5b and S14). Moreover, the stop codon TGA was introduced in the open-reading frame (ORF) of the truncated exon 2 of *AP1*, and TAG was introduced in the ORF of the truncated exon 2 of *TFL1* (Figs. 5b and S15). This result showed that loss of function in *AP1*, *SVP*, and *TFL1* induced by frameshift mutations using the CRISPR/Cas9 system using two sgRNAs was successful in T1 plants.

### Floral characterization of mutant plants

To determine the functions of *AP1*, *SVP*, and *TFL1* in floral development, we analyzed the phenotype of the wild-type, *AP1*-A2, *SVP*-S2, *TFL1*-T1, and triple mutant Tripled-26 plants.

**Fig. 6:**
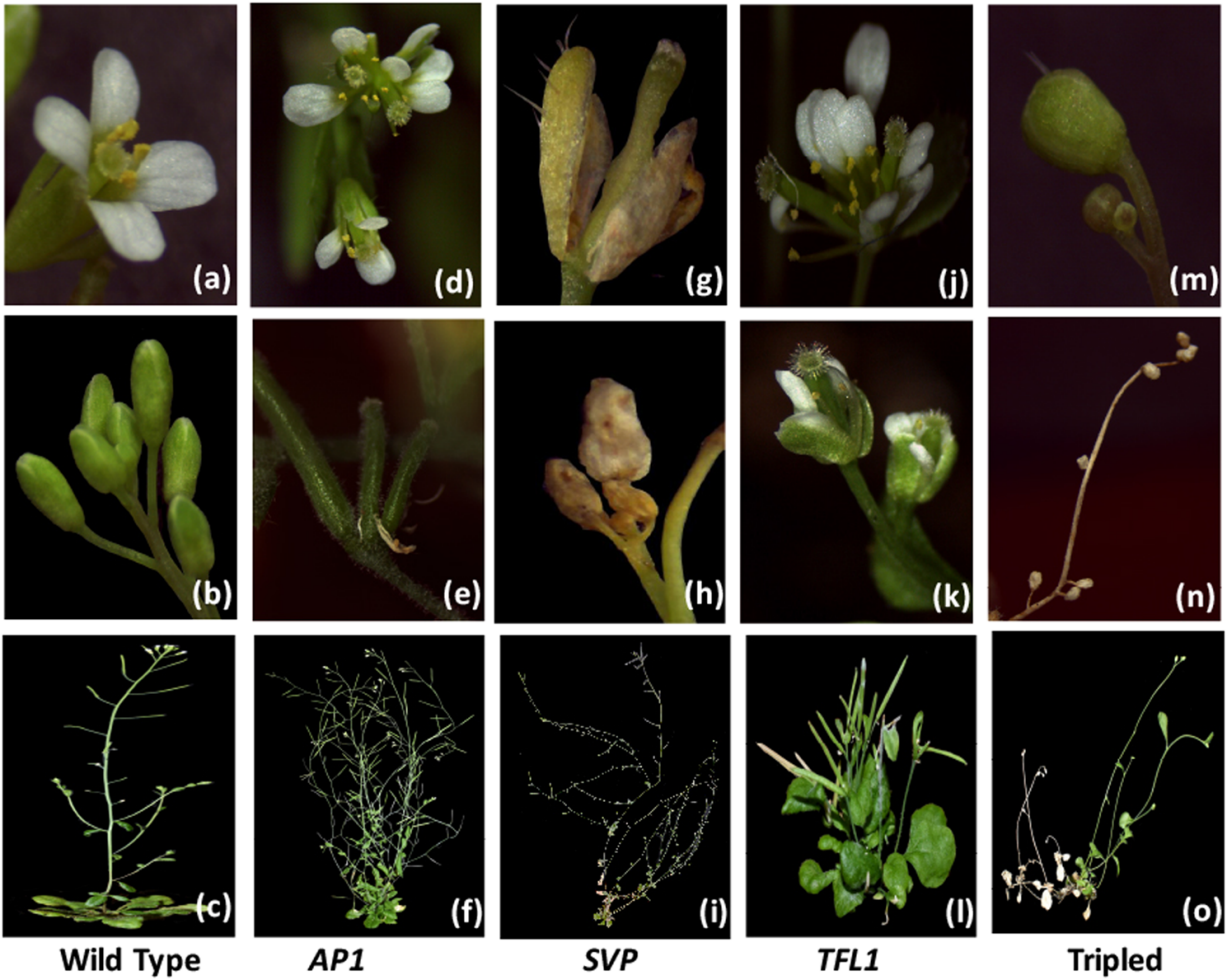
Partial floral development phenotypes of targeted deletion plants. **a**–**c** Wild-type *Arabidopsis*. **d**–**f** *AP1* mutants with strong branching characteristics and doubled or tripled carpels fused together. **g**–**i** *SVP* mutants with increased branching and obvious fourth-order branching showed decreased fruit number and floral abortion. **j**–**l** *TFL1* mutants with dwarf determinate inflorescences, doubled carpels and increased petal numbers. **m**–**o** The floral development of the triple-gene mutant with aborted determinate inflorescences. All plants were grown for 40 days

*AP1* encodes a MADS-domain transcription factor and activates floral organ identity genes to promote floral meristem formation^32^. Targeted deletion of *AP1* resulted in floral meristem development abnormally with an increased number of petals (Fig. 6d) and three degenerated carpels united with sepals and petals (Fig. 6e). It also resulted in a significant increase in plant branching, usually with an additional secondary branch that maintained the indeterminate inflorescence phenotype in the mutant line (Fig. 6f). *SVP* encodes a MADS-domain transcription factor and is a flowering time regulator. However, it acts as a repressor of flowering^33^. The flowering of *SVP* mutants showed early floral meristem formation during the vegetative phase, the inflorescence and flower morphologies were aborted and failed to form pistils and stamens (Fig. 6g), and the flower buds were abnormal (Fig. 6h). There was usually an additional fourth branch growing from the secondary branch (Fig. 6i). *TFL1* is a negative regulator controlling flowering time and inflorescence architecture^34^. *TFL1* mutants showed a partial transformation of the stem meristem into a floral meristem (Fig. 6j), increased the petal number (Fig. 6k) and displayed a change from indeterminate to determinate inflorescence type (Fig. 6k). This result indicates that *TFL1* can not only maintain unlimited inflorescence growth in plants with indeterminate inflorescences but also affect floral development in *Arabidopsis*.

In addition, the absence of *AP1*, *SVP*, and *TFL1* in triple mutant plants caused the continuous production of inflorescence meristem in place of flowers but with the cauliflower phenotype (Fig. 6m). Moreover, some floral meristems were eventually formed and ultimately withered with the entire branch (Fig. 6n). This phenotype is very similar to that of the *SVP* mutant. However, the determinate inflorescence that was formed in the triple mutant was similar to that of the *TFL1* mutant (Fig. 6o). Even if new shoots continued to be produced at the base to maintain the vegetative growth stage, the growth order of *Arabidopsis thaliana* would eventually be broken, resulting in wilting and abortion due to the disorder of the flowering gene network. This result suggests that *AP1*, *SVP*, and *TFL1* together play a role in the regulation of flower formation.

## Discussion

The CRISPR/Cas9 system has great potential for promoting functional studies in plants, as it can be easily adapted to generate loss-of-function mutations for single genes or multiple-gene clusters of unknown function and is becoming one of the most powerful tools for creating functional gene knockouts^2,19,35–37^. However, a major limitation for the use of CRISPR/Cas9 in functional genomic studies in plants is the difficulty of rapidly generating and detecting stable homozygous mutations with high efficiency, as well as the inability to simultaneously mutate multiple target genes^23^. Cas9 most frequently creates a blunt-ended DSB at a position three base pairs upstream of the PAM sequence^7^. Strikingly, we observed a very high frequency of precise ligation in our experiment: on average, 73% of all reads with long deletions were targeted deletions of 1528 bp in *AP1*, 995 bp in *SVP*, or 278 bp in *TFL1*. These results suggest that if a single sgRNA was used, most instances of NHEJ repair would not result in mutation. Therefore, creating deletions with two or more sgRNAs should be a more efficient approach to achieve targeted mutagenesis. Our work demonstrates a suitable approach for targeted loss-of-function deletions by CRISPR/Cas9 in plants.

Because the DSBs generated by two sgRNAs are likely to be accurately repaired, we favor the use of two sgRNAs to induce loss-of-function mutations. Selection of two sgRNAs can be used advantageously to make specific mutations. There are no concerns about creating functional gene isoforms through non-frameshift indels or alternative splicing^19^, and the targeted deletions can be detected by PCR, making screening for mutants significantly easier than when using one sgRNA^15^. In our study, the targeted deletion lines generated by two sgRNAs targeting each gene were detected by PCR analysis. The targeted deletion frequency by the pair of sgRNAs used to create the mutant lines was 30%. In our study, the target sites of *AP1* and *TFL1* showed higher editing efficiencies than the target sites of *SVP*. In contrast to the targeted deletions, gRNA5 and gRNA6 showed very high rates of single-target DSBs in most transgenic-positive lines. Additionally, we found that the GC contents of these sgRNAs ranged from 40% to 60%, which is appropriate for sgRNAs (Table 1). The editing efficiency is limited by the efficiency of the selected target locus, guide RNA structure, sufficient expression of Cas9, and multigenerational analysis of transgenic lines for more multiplexed sgRNA-based editing in plants.

There are different strategies for multiple guide RNA generation. Such polycistronic mRNAs are processed posttranscriptionally into individual sgRNAs by RNA-cleaving enzymes^38^. These enzymes include the CRISPR-associated RNA endoribonuclease Csy4 from *P. aeruginosa*, the tRNA processing enzymes naturally present in the host cells and ribozymes^23,26^. A significant challenge in plant genome engineering is achieving high-frequency genome editing by multiple sgRNAs derived from a polycistronic transcript. Our pDIR21-Triple construct contained six sgRNAs to target the deletion of three genes at the same time with a 4% frequency; it also generated single-gene and double-gene deletions with these multiplexed sgRNAs. We favored the Csy4 system because it consistently gave us higher deletion frequencies in *Arabidopsis*, and the multiple sgRNA transcripts are shorter and less repetitive than those used in the tRNA processing system^26^. Moreover, the polycistronic transcript sgRNAs do not have the limitation of A or G initial nucleotides in sgRNA for the Pol III promoter. There is a report that the CRISPR/Cas9-linked viral silencing suppressor p19 can generate 70% editing efficiency in *Arabidopsis*, but p19 induced a strong leaf developmental phenotype^39^. However, the Csy4 ribonuclease has no observable phenotypic consequences for transgenic plants^24^.

Due to the sequence alterations in the coding region of the target gene induced by the cellular DNA DSB repair mechanism, causing frameshift mutations, the CRISPR/Cas9 system is widely used as an efficient tool for specific gene knockout production. In a recent study, it was shown that indels introduced by CRISPR/Cas9 using one sgRNA can lead to random splicing as opposed to mRNA degradation or protein truncation^40,41^. In our demonstrated method, the cDNA sequence confirmed loss of function in *AP1* and *TFL1* because a stop codon was introduced by a frameshift mutation generated from the targeted deletion of exon 1 and exon 2. Furthermore, the cDNA of *SVP* in the mutant line was not acquired. This result indicated that the targeted deletion between exon 1 and exon 2 of *SVP* significantly decreased mRNA transcription, and thus, this sequence may include promoter fragments and cis-regulatory motifs that change the expression patterns of *SVP*. This result revealed novel insights into the functional mechanisms of divergent flowering-related gene regulation in plants^42^. The exon splicing mechanism reveals the existence of complex gene repair and expression^43,44^. Moreover, there is a report that fertility seemed normal in an *SVP* mutant created by transposon-tagging mutagenesis^45^; however, for *SVP* mutants with either single-gene mutation or triple-gene mutation, deletion of the chromosomal fragment of *SVP* exon 1 and exon 2 by CRISPR/Cas9 using gRNA3/gRNA4 of pDIR21-*SVP* or pDIR21-Triple directly affected the fruiting of the resultant T1 plants. Detailed studies on the differences in these parameters will require further experimentation to fully test.

## Materials and methods

### Plant material

*Arabidopsis thaliana* Col-0 plants were grown in a growth chamber that was set at 16/8 h (light/dark) and 22 °C. T1 seeds derived from plants transformed with binary vectors were selected on B5 media lacking sucrose and containing 30 mg/L hygromycin B.

### Vector construction

The plasmid pDIRECT-21 purchased from ATCG (http://www.addgene.org/91130) was used as a backbone for the construction of the Cas9-sgRNA-expressing vector. Briefly, the Cas9 gene sequence, including the nuclear localization signals, was designed with codon optimization for dicot plants, and the Csy4 RNA nuclease was fused into the N terminus of Cas9 splitting by P2A. The 35s promoter was used to express the Csy4-Cas9 module, and the CmYLCV promoter drove expression of the sgRNA processed by Csy4 to release the multiplexed sgRNAs. Efficient protospacer sequences targeting the coding exons of *AP1*, *SVP*, and *TFL1* were selected using the Zhang Laboratory CRISPR Design website (http://crispr.mit.edu). Oligonucleotides corresponding to the top and bottom strands of the sgRNAs were synthesized, annealed, and cloned into pDIRECT-21 as previously described^24^ to generate four different binary vectors encoding the Cas9 nuclease and sgRNAs. These vectors were used for *Agrobacterium*-mediated *Arabidopsis* transformation after DNA sequencing confirmation.

The genome-editing vector set uses Gibson assembly cloning to create vectors for a diverse range of genome-editing applications. There are three sets of modular cloning fragments. Fragment A contains the CmYLCV promoter and was amplified by the primers CmYLCV-F/CmYLCV-RA. Fragment B contains multiple sgRNAs targeting *AP1* sites and was produced by annealing the oligos gRNA1-F/gRNA1-R or gRNA2-F/gRNA-R separately. Fragment C contains the backbone of pDIRECT-21 digested by SapI restriction enzyme. These fragments were assembled using Gibson assembly cloning kits (NEB, USA) to construct pDIR21-*AP1*. Using the same method, we acquired pDIR21-*SVP* by CmYLCV-F/ CmYLCV-RS gRNA3-F/gRNA3-R and gRNA4-F/gRNA-R and pDIR21-*TFL1* by CmYLCV-F/ CmYLCV-RT, gRNA5-F/gRNA5-R, and gRNA6-F/gRNA-R separately. For pDIR21-Triple, six sgRNAs were assembled by gRNA1-F/gRNA-triple-R1, gRNA3-F/gRNA-triple-R2, gRNA5-F/gRNA-triple-R3, gRNA2-F/gRNA-triple-R4, gRNA4-F/gRNA-triple-R5, and gRNA6-F/gRNA-R separately.

### Agrobacterium-mediated *Arabidopsis* transformation

Genetic transformation of *Arabidopsis* was conducted using the common floral dip method^46^. Wild-type (WT) Col-0 plants with flower buds were infected with activated *Agrobacterium tumefaciens* EHA105. The seeds from the infected plants were collected, dried, surface-disinfected with 70% ethanol for 10 min, and rinsed with 90% ethanol once and autoclaved ddH_2_O three times. The seeds were then cultured on agar-solidified half-strength Murashige and Skoog medium^47^ containing 30 mg/L hygromycin. Seed vernalization was performed at 4 °C for 3 days in the dark. Germination was performed under a photoperiod of 16/8 h (light/dark) at 22 °C for 1 day, followed by 3 days in the dark and then 3 days with a photoperiod of 16/8 (light/dark) at 22 °C. Hygromycin B-resistant seedlings screened from the dishes were transplanted onto soil for continuous growth to allow molecular analysis, phenotype observation, and seed harvesting.

### Genomic DNA extraction and PCR analysis

Genomic DNA was extracted from *Arabidopsis* leaves using the CTAB method following the published protocol^48^. To detect mutagenesis at the desired sites, the target regions were amplified with specific primers (see supplementary information for primer sequences) using Premix Taq DNA Polymerase (Takara, Japan) with the following protocol: 94 °C for 10 min (94 °C for 30 s, 60–48 °C for 30 s, 72 °C for 2 min) for 13 cycles with touchdown −1 °C in each cycle (94 °C for 30 s, 52 °C for 30 s, 72 °C for 2 min) for 25 cycles, 72 °C for 10 min, 4 °C to hold. The PCR product was separated in a 1% agarose gel and stained with GelStain (TransGen Biotech, China) to detect chromosomal fragment deletions. Selected PCR products were cloned into the pGEM-T Easy Vector (Promega, USA) for Sanger DNA sequencing.

### RNA extraction and RT-PCR

Total RNA was extracted using the NucleoSpin RNA Plant Kit (Takara), treated with DNase before use as the template for RT-PCR, analyzed in a 1.2% agarose gel and stained with GelStain (TransGen Biotech, China) to assess the extracted total RNA concentration and integrity. Additionally, no degradation was found in the RNA extracts, as 18S:28S was equal to 1:2 for all samples. cDNA was then generated by reverse transcription from 1 µg of total RNA using 25 U of AMV reverse transcriptase, 100 mM dNTPs, 25 U of RNase inhibitor and 100 µm Oligo-d(T)_18_ Primers (AMV Reverse Transcriptase Kit, Promega, USA) in a 25 µl reaction volume. The reverse transcription reaction was carried out in three steps: 60 min at 42 °C, 30 min at 55 °C and 15 min at 70 °C. PCRs were performed with 1 µl of cDNA using Premix Taq DNA Polymerase (Takara, Japan). The PCR products were separated in a 1% agarose gel and stained with GelStain (TransGen Biotech, China) to detect the cDNA of the target gene. Selected PCR products were cloned into pGEM-T Easy Vector (Promega, USA) for DNA sequencing.

### Gene accession numbers in GenBank

The genes and their GenBank RefSeq accession numbers are as follows: *AP1*, NC_003070.9; *SVP*, NC_003071.7; *TFL1*, NC_003076.8.

## Supplementary information

Supplementary information

## References

[1] Liu D, Mewalal R, Hu R, Tuskan GA, Yang X (2017). New technologies accelerate the exploration of non-coding RNAs in horticultural plants. Hortic. Res..

[2] Meng X (2017). Construction of a Genome-Wide Mutant Library in Rice using CRISPR/Cas9. Mol. Plant.

[3] Samanta MK, Dey A, Gayen S (2016). CRISPR/Cas9: an advanced tool for editing plant genomes. J. Transgenic Res..

[4] Voytas DF, Gao C (2014). Precision genome engineering and agriculture: opportunities and regulatory challenges. PLOS Biol..

[5] Yin K, Gao C, Qiu J-L (2017). Progress and prospects in plant genome editing. Nat. Plants.

[6] Cong L (2013). Multiplex genome engineering using CRISPR/Cas systems. Science.

[7] Jinek M (2012). A programmable Dual-RNA-guided DNA. Science.

[8] Mali P (2013). RNA-guided human genome engineering via Cas9. Science.

[9] Durr J, Papareddy R, Nakajima K, Gutierrez-Marcos J (2018). Highly efficient heritable targeted deletions of gene clusters and non-coding regulatory regions in *Arabidopsis* using CRISPR/Cas9. Sci. Rep..

[10] Feng Z (2014). Multigeneration analysis reveals the inheritance, specificity, and patterns of CRISPR/Cas-induced gene modifications in *Arabidopsis*. Proc. Natl Acad. Sci. USA.

[11] Gao X, Chen J, Dai X, Zhang D, Zhao Y (2016). An effective strategy for reliably isolating heritable and Cas9-free *Arabidopsis* mutants generated by CRISPR/Cas9-mediated genome editing. Plant Physiol..

[12] Ma X, Zhu Q, Chen Y, Liu Y-G (2016). CRISPR/Cas9 platforms for genome editing in plants: developments and applications. Mol. Plant.

[13] Osakabe, Y. & Osakabe, K. in *Progress in Molecular Biology and Translational Science,* Vol. 149 (eds Weeks, Donald P. & Yang, Bing) 99–109 (Academic Press, 2017).

[14] Chen L (2018). A method for the production and expedient screening of CRISPR/Cas9-mediated non-transgenic mutant plants. Hortic. Res..

[15] Li J-F (2013). Multiplex and homologous recombination–mediated genome editing in *Arabidopsis* and *Nicotiana benthamiana* using guide RNA and Cas9. Nat. Biotechnol..

[16] Ma X (2015). A robust CRISPR/Cas9 system for convenient, high-efficiency multiplex genome editing in monocot and dicot plants. Mol. Plant.

[17] Mao Y (2016). Development of germ-line-specific CRISPR-Cas9 systems to improve the production of heritable gene modifications in *Arabidopsis*. Plant Biotechnol. J..

[18] Gao J (2015). CRISPR/Cas9-mediated targeted mutagenesis in Nicotiana tabacum. J. Plant Mol. Biol..

[19] Lalonde S (2017). Frameshift indels introduced by genome editing can lead to in-frame exon skipping. PLoS ONE.

[20] Nishitani C (2016). Efficient genome editing in apple using a CRISPR/Cas9 system. Sci. Rep..

[21] Zhang F, Wen Y, Guo X (2014). CRISPR/Cas9 for genome editing: progress, implications and challenges. Hum. Mol. Genet..

[22] Yu Zhiming, Chen Qiyuan, Chen Weiwei, Zhang Xian, Mei Fengling, Zhang Pengcheng, Zhao Mei, Wang Xiaohong, Shi Nongnong, Jackson Stephen, Hong Yiguo (2018). Multigene editing via CRISPR/Cas9 guided by a single-sgRNA seed in Arabidopsis. Journal of Integrative Plant Biology.

[23] Gao Y, Zhao Y (2014). Self-processing of ribozyme-flanked RNAs into guide RNAs in vitro and in vivo for CRISPR-mediated genome editing. J. Integr. Plant Biol..

[24] Čermák T (2017). A multipurpose toolkit to enable advanced genome engineering in plants. Plant Cell.

[25] Tsai SQ (2014). Dimeric CRISPR RNA-guided FokI nucleases for highly specific genome editing. Nat. Biotechnol..

[26] Xie K, Minkenberg B, Yang Y (2015). Boosting CRISPR/Cas9 multiplex editing capability with the endogenous tRNA-processing system. Proc. Natl Acad. Sci. USA.

[27] Blázquez MA (2005). The right time and place for making flowers. Science.

[28] Jaeger KE, Pullen N, Lamzin S, Morris RJ, Wigge PA (2013). Interlocking feedback loops govern the dynamic behavior of the floral transition in *Arabidopsis*. J. Plant Cell.

[29] Liu J (2018). MIKC(C)-type MADS-box genes in *Rosa chinensis*: the remarkable expansion of ABCDE model genes and their roles in floral organogenesis. Hortic. Res..

[30] Liu C (2013). A conserved genetic pathway determines inflorescence architecture in *Arabidopsis* and Rice. Dev. Cell.

[31] Baumann K (2015). Changing the spatial pattern of TFL1 expression reveals its key role in the shoot meristem in controlling *Arabidopsis* flowering architecture. J. Exp. Bot..

[32] Kempin S, Savidge B, Yanofsky M (1995). Molecular basis of the cauliflower phenotype in *Arabidopsis*. Science.

[33] Gregis V (2013). Identification of pathways directly regulated by SHORT VEGETATIVE PHASE during vegetative and reproductive development in *Arabidopsis*. Genome Biol..

[34] Hanano S, Goto K (2011). Arabidopsis TERMINAL FLOWER1 is involved in the regulation of flowering time and inflorescence development through transcriptional repression. J. Plant Cell.

[35] Agarwal A (2018). Insights into maize genome editing via CRISPR/Cas9. Physiol. Mol. Biol. Plants.

[36] Demirci Y, Zhang B, Unver T (2018). CRISPR/Cas9: an RNA-guided highly precise synthetic tool for plant genome editing. J. Cell Physiol..

[37] Hussain B, Lucas SJ, Budak H (2018). CRISPR/Cas9 in plants: at play in the genome and at work for crop improvement. Brief. Funct. Genom..

[38] He, Y., Wang, R., Dai, X. & Zhao, Y. in *Progress in Molecular Biology and Translational Science* Vol. 149 (eds Weeks, Donald P. & Yang, Bing) 151-166 (Academic Press, 2017).

[39] Mao Y (2018). Manipulating plant RNA-silencing pathways to improve the gene editing efficiency of CRISPR/Cas9 systems. J. Genome Biol..

[40] Chen D (2018). CRISPR/Cas9-mediated genome editing induces exon skipping by complete or stochastic altering splicing in the migratory locust. BMC Biotechnol..

[41] Kapahnke M, Banning A, Tikkanen R (2016). Random splicing of several exons caused by a single base change in the target exon of CRISPR/Cas9 mediated gene knockout. Cells.

[42] Dhadi SR, Xu Z, Shaik R, Driscoll K, Ramakrishna W (2015). Differential regulation of genes by retrotransposons in rice promoters. J. Plant Mol. Biol..

[43] Mao Y (2016). Functional analysis of alternative splicing of the FLOWERING LOCUS T orthologous gene in *Chrysanthemum morifolium*. Hortic. Res..

[44] Xue Chenxiao, Zhang Huawei, Lin Qiupeng, Fan Rong, Gao Caixia (2018). Manipulating mRNA splicing by base editing in plants. Science China Life Sciences.

[45] Gregis V, Sessa A, Colombo L, Kater MM (2008). AGAMOUS-LIKE24 and SHORT VEGETATIVE PHASE determine floral meristem identity in *Arabidopsis*. Plant J..

[46] Clough SJ, Bent AF (1998). Floral dip: a simplified method for Agrobacterium-mediated transformation of *Arabidopsis thaliana*. Plant J..

[47] Murashige T, Skoog R (1962). A revised medium for rapid growth and bio assays with tobacco tissue cultures. Physiol. Plant.

[48] Healey A, Furtado A, Cooper T, Henry RJ (2014). Protocol: a simple method for extracting next-generation sequencing quality genomic DNA from recalcitrant plant species. Plant Methods.

