# Supplementary material for: Targeted deletion of floral development genes in Arabidopsis with CRISPR/Cas9 using the RNA endoribonuclease Csy4 processing system
Source: Hortic Res. 2019 Aug 21;6:99. doi: 10.1038/s41438-019-0179-6 (PMC6804923; doi:10.1038/s41438-019-0179-6)

**Supplementary information**

1. **Sequence of multiplexed sgRNAs for genome editing.**

**gRNA cassette of pDIR21-*AP1***

tggcagacatactgtcccacaaatgaagatggaatctgtaaaagaaaacgcgtgaaataatgcgtctgacaaaggttaggtcggctgcctttaatcaataccaaagtggtccctaccacgatggaaaaactgtgcagtcggtttggctttttctgacgaacaaataagattcgtggccgacaggtgggggtccaccatgtgaaggcatcttcagactccaataatggagcaatgacgtaagggcttacgaaataagtaagggtagtttgggaaatgtccactcacccgtcagtctataaatacttagcccctccctcattgttaagggagcaaaatctcagagagatagtcctagagagagaaagagagcaagtagcctagaagtagtcaaggcggcgaagtattcaggcacgtggccaggaagaagaaaagccaagacgacgaaaacaggtaagagctaagcttcctgcaggttcactgccgtataggcagGGGGTAGGGTTCAATTGAAG**gttttagagctagaaatagcaagttaaaataaggctagtccgttatcaacttgaaaaagtggcaccgagtcggtgc**gttcactgccgtataggcagAGATACTTGAACGCTATGAG**gttttagagctagaaatagcaagttaaaataaggctagtccgttatcaacttgaaaaagtggcaccgagtcggtgc**gttcactgccgtataggcaggctcttcagtcgatcgacaagctcgagtttctccataataatgtgtgagtagttcccagataagggaattagggttcctatagggtttcgctcatgtgttgagcatataagaaacccttagtatgtatttgtatttgtaaaatacttctatcaataaaatttctaattcctaaaaccaaaatccagtactaaaatccagatc

**gRNA cassette of pDIR21-*SVP***

tggcagacatactgtcccacaaatgaagatggaatctgtaaaagaaaacgcgtgaaataatgcgtctgacaaaggttaggtcggctgcctttaatcaataccaaagtggtccctaccacgatggaaaaactgtgcagtcggtttggctttttctgacgaacaaataagattcgtggccgacaggtgggggtccaccatgtgaaggcatcttcagactccaataatggagcaatgacgtaagggcttacgaaataagtaagggtagtttgggaaatgtccactcacccgtcagtctataaatacttagcccctccctcattgttaagggagcaaaatctcagagagatagtcctagagagagaaagagagcaagtagcctagaagtagtcaaggcggcgaagtattcaggcacgtggccaggaagaagaaaagccaagacgacgaaaacaggtaagagctaagcttcctgcaggttcactgccgtataggcagGACATCGGCGTCGCAGAGAA**gttttagagctagaaatagcaagttaaaataaggctagtccgttatcaacttgaaaaagtggcaccgagtcggtgc**gttcactgccgtataggcagACTGCAAGTTATGCCTCTCT**gttttagagctagaaatagcaagttaaaataaggctagtccgttatcaacttgaaaaagtggcaccgagtcggtgc**gttcactgccgtataggcaggctcttcagtcgatcgacaagctcgagtttctccataataatgtgtgagtagttcccagataagggaattagggttcctatagggtttcgctcatgtgttgagcatataagaaacccttagtatgtatttgtatttgtaaaatacttctatcaataaaatttctaattcctaaaaccaaaatccagtactaaaatccagatc

**gRNA cassette of pDIR21-*TFL1***

tggcagacatactgtcccacaaatgaagatggaatctgtaaaagaaaacgcgtgaaataatgcgtctgacaaaggttaggtcggctgcctttaatcaataccaaagtggtccctaccacgatggaaaaactgtgcagtcggtttggctttttctgacgaacaaataagattcgtggccgacaggtgggggtccaccatgtgaaggcatcttcagactccaataatggagcaatgacgtaagggcttacgaaataagtaagggtagtttgggaaatgtccactcacccgtcagtctataaatacttagcccctccctcattgttaagggagcaaaatctcagagagatagtcctagagagagaaagagagcaagtagcctagaagtagtcaaggcggcgaagtattcaggcacgtggccaggaagaagaaaagccaagacgacgaaaacaggtaagagctaagcttcctgcaggttcactgccgtataggcagCTTCTGTTTCCTCCAAGCCT**gttttagagctagaaatagcaagttaaaataaggctagtccgttatcaacttgaaaaagtggcaccgagtcggtgc**gttcactgccgtataggcagATGATAGACCCAGATGTTCC**gttttagagctagaaatagcaagttaaaataaggctagtccgttatcaacttgaaaaagtggcaccgagtcggtgc**gttcactgccgtataggcaggctcttcagtcgatcgacaagctcgagtttctccataataatgtgtgagtagttcccagataagggaattagggttcctatagggtttcgctcatgtgttgagcatataagaaacccttagtatgtatttgtatttgtaaaatacttctatcaataaaatttctaattcctaaaaccaaaatccagtactaaaatccagatc

**gRNA cassette of pDIR21-Triple**

tggcagacatactgtcccacaaatgaagatggaatctgtaaaagaaaacgcgtgaaataatgcgtctgacaaaggttaggtcggctgcctttaatcaataccaaagtggtccctaccacgatggaaaaactgtgcagtcggtttggctttttctgacgaacaaataagattcgtggccgacaggtgggggtccaccatgtgaaggcatcttcagactccaataatggagcaatgacgtaagggcttacgaaataagtaagggtagtttgggaaatgtccactcacccgtcagtctataaatacttagcccctccctcattgttaagggagcaaaatctcagagagatagtcctagagagagaaagagagcaagtagcctagaagtagtcaaggcggcgaagtattcaggcacgtggccaggaagaagaaaagccaagacgacgaaaacaggtaagagctaagcttcctgcaggttcactgccgtataggcagGGGGTAGGGTTCAATTGAAG**gttttagagctagaaatagcaagttaaaataaggctagtccgttatcaacttgaaaaagtggcaccgagtcggtgc**gttcactgccgtataggcagGACATCGGCGTCGCAGAGAA**gttttagagctagaaatagcaagttaaaataaggctagtccgttatcaacttgaaaaagtggcaccgagtcggtgc**gttcactgccgtataggcagCTTCTGTTTCCTCCAAGCCT**gttttagagctagaaatagcaagttaaaataaggctagtccgttatcaacttgaaaaagtggcaccgagtcggtgc**gttcactgccgtataggcagAGATACTTGAACGCTATGAG**gttttagagctagaaatagcaagttaaaataaggctagtccgttatcaacttgaaaaagtggcaccgagtcggtgc**gttcactgccgtataggcagACTGCAAGTTATGCCTCTCT**gttttagagctagaaatagcaagttaaaataaggctagtccgttatcaacttgaaaaagtggcaccgagtcggtgc**gttcactgccgtataggcagATGATAGACCCAGATGTTCC**gttttagagctagaaatagcaagttaaaataaggctagtccgttatcaacttgaaaaagtggcaccgagtcggtgc**gttcactgccgtataggcaggctcttcagtcgatcgacaagctcgagtttctccataataatgtgtgagtagttcccagataagggaattagggttcctatagggtttcgctcatgtgttgagcatataagaaacccttagtatgtatttgtatttgtaaaatacttctatcaataaaatttctaattcctaaaaccaaaatccagtactaaaatccagatc

Dark is CmYLCV promoter and CaMV poly(A) signal sequence.

Underline is 20bp Csy4 recognition site.

Capital is 20bp protospacer sequence.

Bold is gRNA scaffold sequence.

**Supplementary Table 1. Primer list**

| **Primer ID** | **Sequence (5’-3’)** |
| --- | --- |
| AP1-1 | GATCCCGAGACTCAAACACAAG |
| AP1-2 | TGGAAATGCTTCATGCGGCGAAGC |
| AP1-3 | GAGTGTAAATGTGTACCTCTGG |
| AP1-4 | GAAGTGAACCCTAGAACACAC |
| AP1-5 | CATTATGAGTTAGGGATCAAAAC |
| AP1-6 | GCTCCAACAAGCAACAGCTGTTC |
| AP1-7 | GATACCTTTATAATAACAAAACA |
| SVP-1 | GATCCATCAAAATCAATCCCGT |
| SVP-2 | GAGAATTCACTACTTAGACATTGTCTC |
| SVP-3 | CTGTTCTCAACCAGCTAACAG |
| SVP-4 | CCATAAATGACATATATAAAGTG |
| SVP-5 | GACATTGACGGCTTAAACATATC |
| SVP-6 | GAGAGATATTATTGATATTAG |
| SVP-7 | CGTTTTCAGTTACATATATACAC |
| TFL-1 | AAGTTAACAAAAGAAAATGGAG |
| TFL-2 | CAGAACACAAATAAACATGAG |
| TFL-3 | TTACCAAACGTAGCATCTGTTG |
| TFL-4 | GGCCATGAGCTCTTTCCTTCTTC |
| TFL-5 | GGCACAATATAAATAAAACCAAC |
| TFL-6 | CGTCTCACTTCCTTTTCCTCTT |
| TFL-7 | GATAAAAACTAAAGCTACAATTTA |
| actin-F | GGCTGGATTTGCAGGAGAGATGATG |
| actin-R | AACGACCTTAATCTTCATGCTGC |
| CmYLCV-F | CGGACGGCGCGCCGAAGAGCTGGCAGACATACTGTCCCACAAATG |
| CmYLCV-RA | ***CTTCAATTGAACCCTACCCC***CTGCCTATACGGCAGTGAACCTGCAGGAAGCTTAGCTCTTACCTG |
| gRNA1-F | **GGGGTAGGGTTCAATTGAAG**GTTTTAGAGCTAGAAATAGCAAGTTAAAATAAGGCTAGTCCGTTATCAACTTGAAA |
| gRNA1-R | ***CTCATAGCGTTCAAGTATCT***CTGCCTATACGGCAGTGAACGCACCGACTCGGTGCCACTTTTTCAAGTTGATAACG |
| gRNA2-F | **AGATACTTGAACGCTATGAG**GTTTTAGAGCTAGAAATAGCAAGTTAAAATAAGGCTAGTCCGTTATCAACTTGAAA |
| CmYLCV-RS | ***TTCTCTGCGACGCCGATGTC***CTGCCTATACGGCAGTGAACCTGCAGGAAGCTTAGCTCTTACCTG |
| gRNA3-F | **GACATCGGCGTCGCAGAGAA**GTTTTAGAGCTAGAAATAGCAAGTTAAAATAAGGCTAGTCCGTTATCAACTTGAAA |
| gRNA3-R | ***AGAGAGGCATAACTTGCAGT***CTGCCTATACGGCAGTGAACGCACCGACTCGGTGCCACTTTTTCAAGTTGATAACG |
| gRNA4-F | **ACTGCAAGTTATGCCTCTCT**GTTTTAGAGCTAGAAATAGCAAGTTAAAATAA**G**GCTAGTCCGTTATCAACTTGAAA |
| CmYLCV-RT | ***AGGCTTGGAGGAAACAGAAG***CTGCCTATACGGCAGTGAACCTGCAGGAAGCTTAGCTCTTACCTG |
| gRNA5-F | **CTTCTGTTTCCTCCAAGCCT**GTTTTAGAGCTAGAAATAGCAAGTTAAAATAAGGCTAGTCCGTTATCAACTTGAAA |
| gRNA5-R | ***GGAACATCTGGGTCTATCAT***CTGCCTATACGGCAGTGAACGCACCGACTCGGTGCCACTTTTTCAAGTTGATAACG |
| gRNA6-F | **ATGATAGACCCAGATGTTCC**GTTTTAGAGCTAGAAATAGCAAGTTAAAATAAGGCTAGTCCGTTATCAACTTGAAA |
| gRNA-triple-R1 | ***TTCTCTGCGACGCCGATGTC***CTGCCTATACGGCAGTGAACGCACCGACTCGGTGCCACTTTTTCAAGTTGATAACG |
| gRNA-triple-R2 | ***AGGCTTGGAGGAAACAGAAG***CTGCCTATACGGCAGTGAACGCACCGACTCGGTGCCACTTTTTCAAGTTGATAACG |
| gRNA-triple-R3 | ***CTCATAGCGTTCAAGTATCT***CTGCCTATACGGCAGTGAACGCACCGACTCGGTGCCACTTTTTCAAGTTGATAACG |
| gRNA-triple-R4 | ***AGAGAGGCATAACTTGCAGT***CTGCCTATACGGCAGTGAACGCACCGACTCGGTGCCACTTTTTCAAGTTGATAACG |
| gRNA-triple-R5 | ***GGAACATCTGGGTCTATCAT***CTGCCTATACGGCAGTGAACGCACCGACTCGGTGCCACTTTTTCAAGTTGATAACG |
| gRNA-R | TTGTCGATCGACTGAAGAGCCTGCCTATACGGCAGTGAACGCACCGACTCGGTGCCACTTTTTCAAGTTG |

Bold is sequence of gRNA1-6 respectively.

Italic Bold is reverse sequence of gRNA1-6 respectively.

**Supplementary Figure 1. DNA sequence of targeted deletion of *AP1* by sgRNA1 and sgRNA2.** Red lines of two nucleotides are junction.


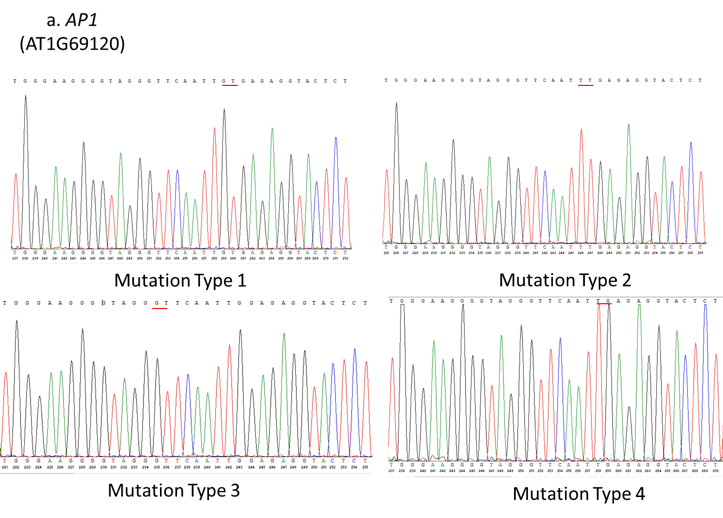


**Supplementary Figure 2. DNA sequence of targeted deletion of *SVP* by sgRNA3 and sgRNA4.** Red lines of two nucleotides are junction.


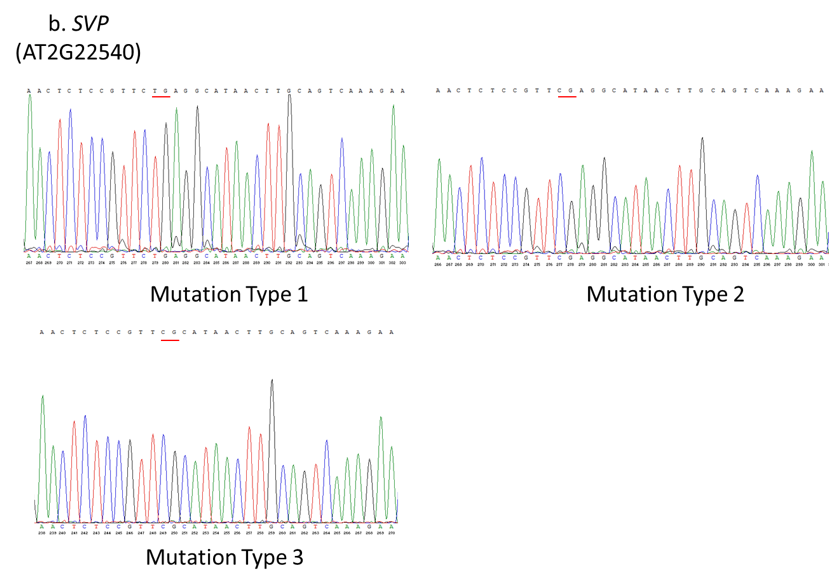


**Supplementary Figure 3. DNA sequence of targeted deletion of *TFL1* by sgRNA5 and sgRNA6.** Red lines of two nucleotides are junction.


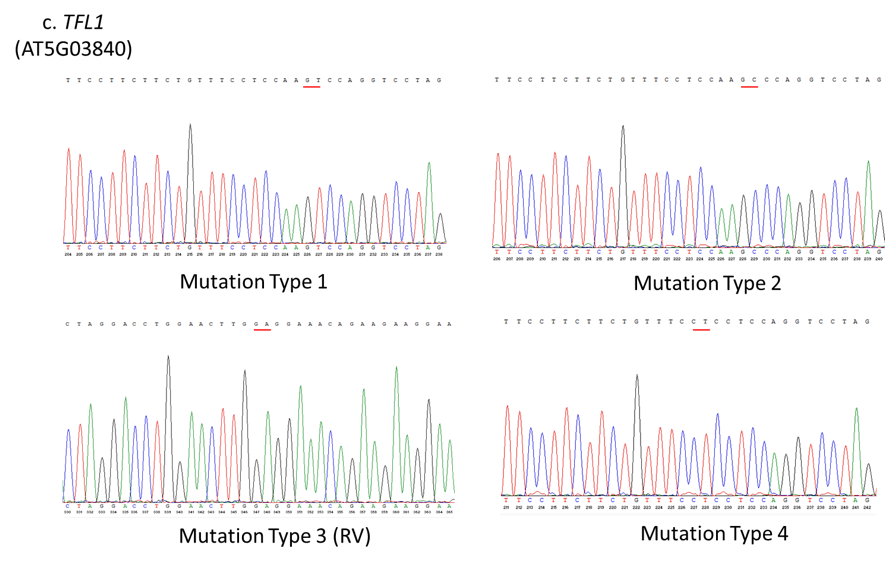


**Supplementary Figure 4. PCR detection of targeted deletion in *AP1* by pDIR21-Triple.** a pair of primer AP-1/AP-3.

**Supplementary Figure 5. PCR detection of targeted deletion in *SVP* by pDIR21-Triple.** A pair of primer SVP-1/SVP-3.

**Supplementary Figure 6. PCR detection of targeted deletion in TFL1 by pDIR21-Triple.** A pair of primer TFL-1/TFL-3.

**Supplementary Figure 7. DNA sequence analysis sgRNA1 single site mutation.** sgRNA target sites is underlined and PAM sequences are in bold. Deleted sequences are in dot. Insertion nucleotides are in italic.

**
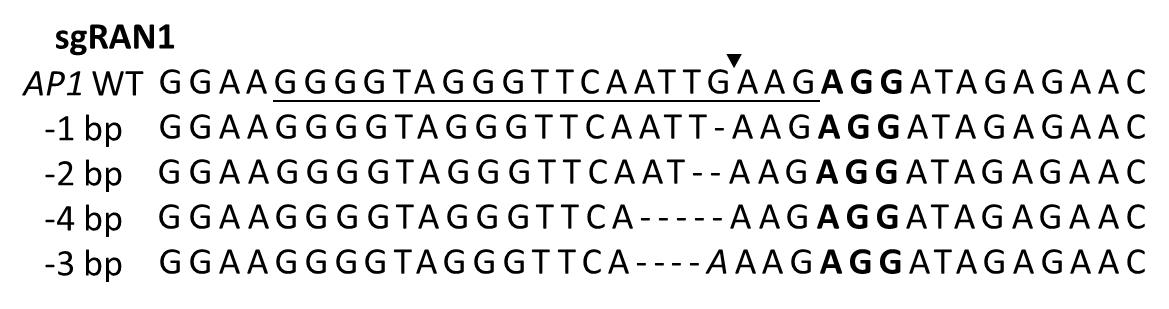
**


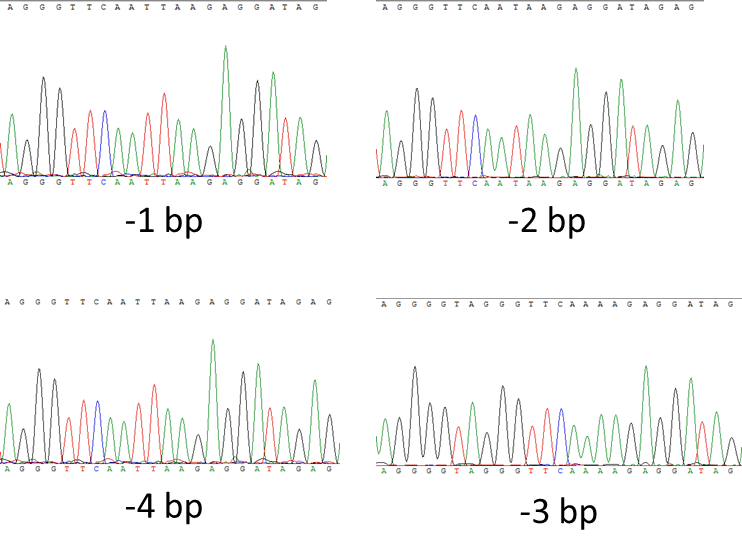


**Supplementary Figure 8. DNA sequence analysis sgRNA2 single site mutation.** sgRNA target sites is underlined and PAM sequences are in bold. Deleted sequences are in dot. Insertion nucleotides are in italic.


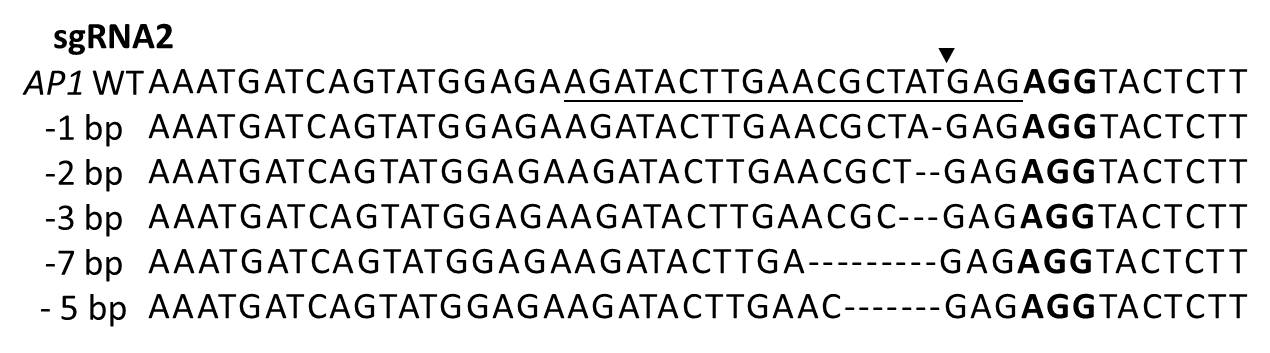


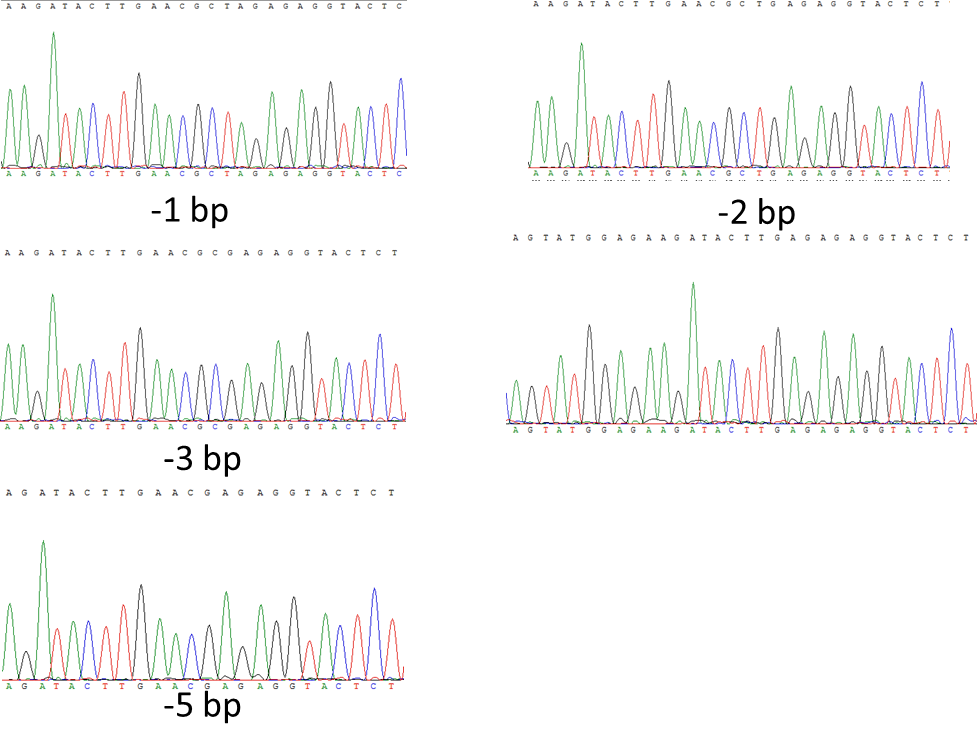


**Supplementary Figure 9. DNA sequence analysis sgRNA4 single site mutation.** sgRNA target sites is underlined and PAM sequences are in bold. Deleted sequences are in dot. Insertion nucleotides are in italic.


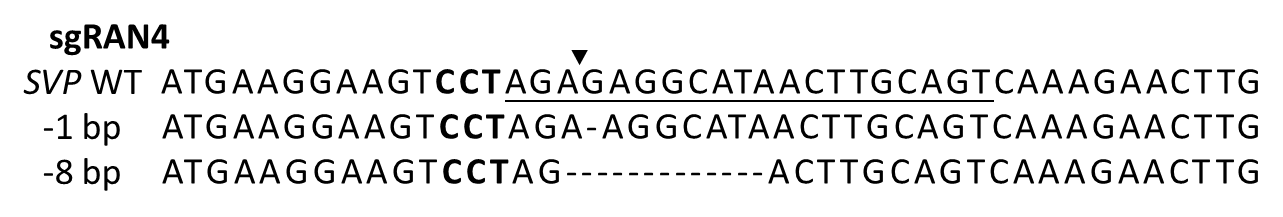


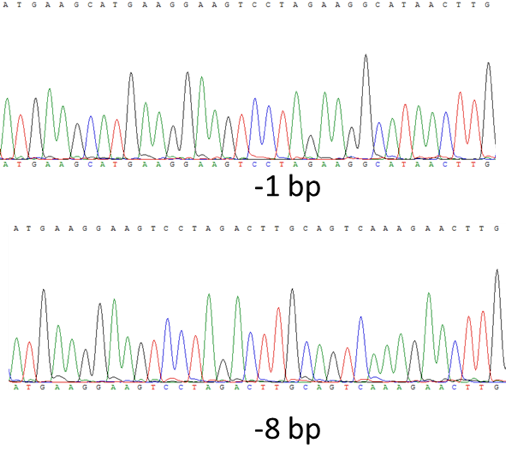


**Supplementary Figure 10. DNA sequence analysis sgRNA5 single site mutation.** sgRNA target sites is underlined and PAM sequences are in bold. Deleted sequences are in dot. Insertion nucleotides are in italic.


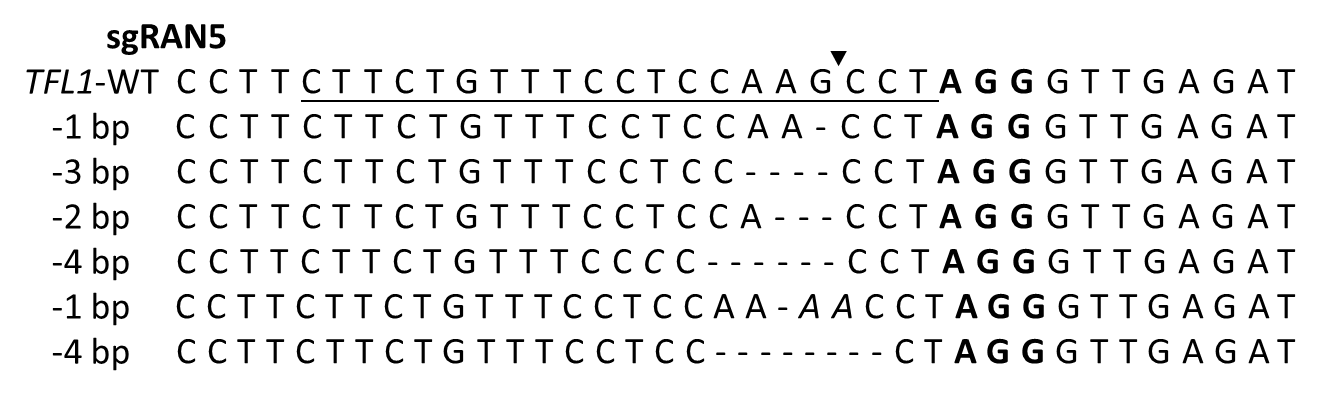


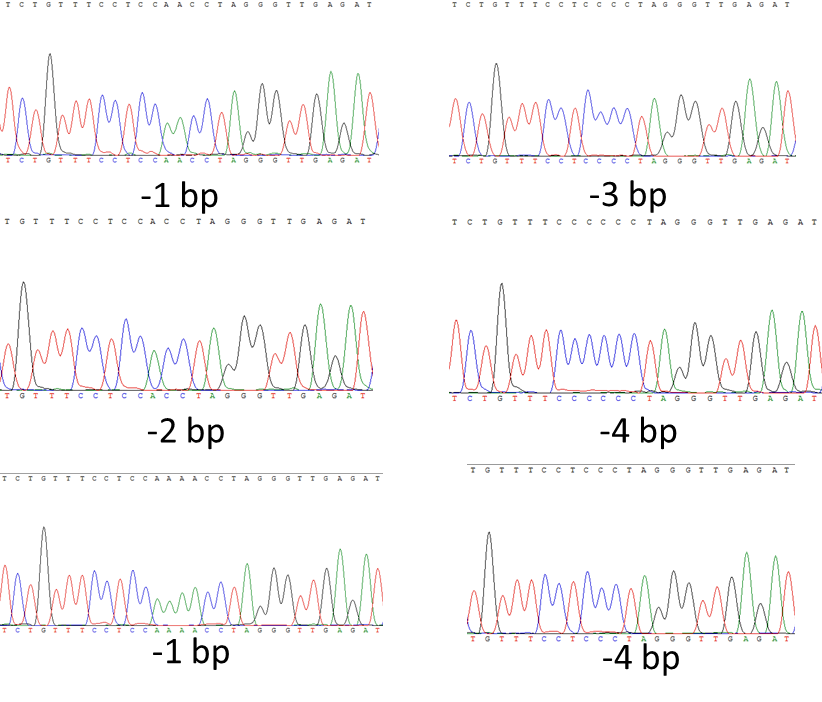


**Supplementary Figure 11. DNA sequence analysis sgRNA6 single site mutation.** sgRNA target sites is underlined and PAM sequences are in bold. Deleted sequences are in dot. Insertion nucleotides are in italic.


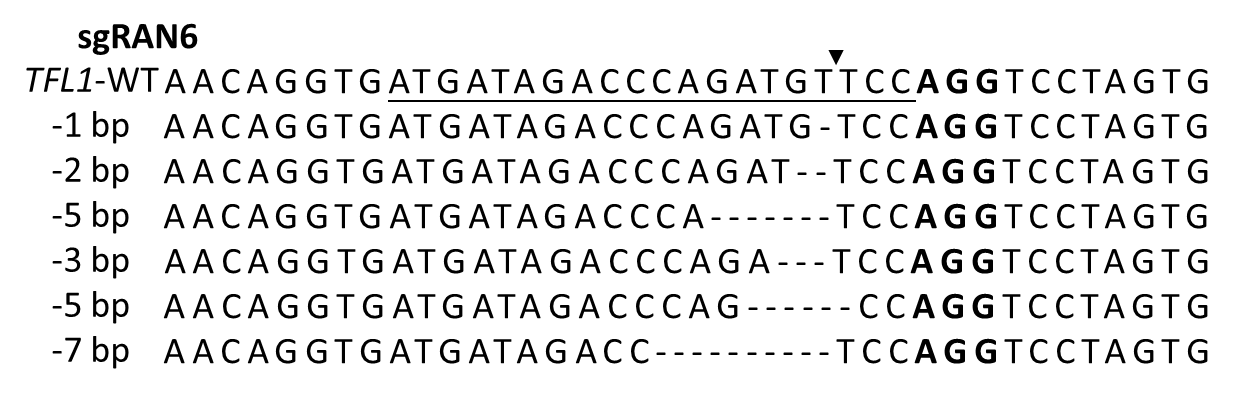


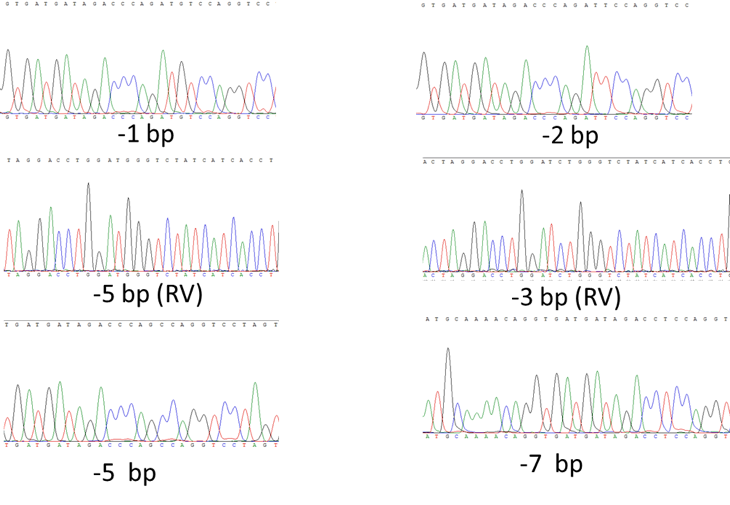


**Supplementary Figure 12. Inversion mutation of sgRNA1 and sgRNA2 in *AP1*.** sgRNA target sites is underlined and PAM sequences are in bold. Deleted sequences are in dot. Inversion nucleotides are in dark.


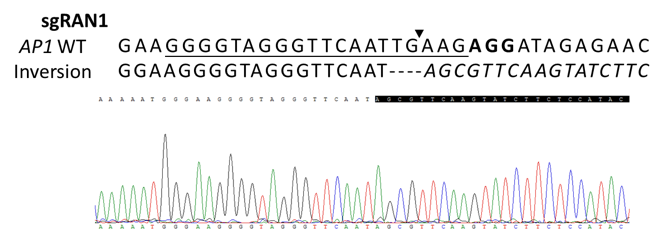


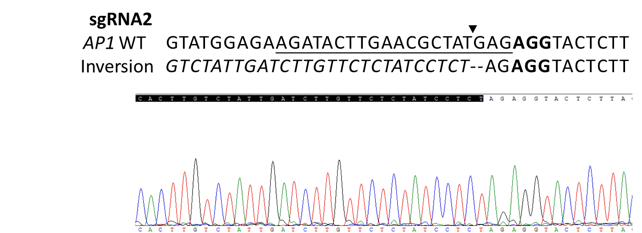


**Supplementary Figure 13. Inversion mutation of sgRNA5 and sgRNA6 in *TFL1*.** sgRNA target sites is underlined and PAM sequences are in bold. Deleted sequences are in dot. Inversion nucleotides are in dark.

**
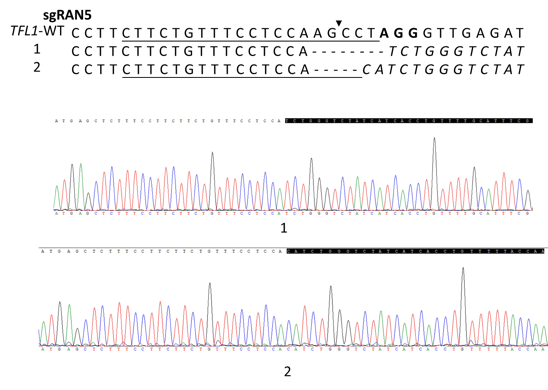
**

**
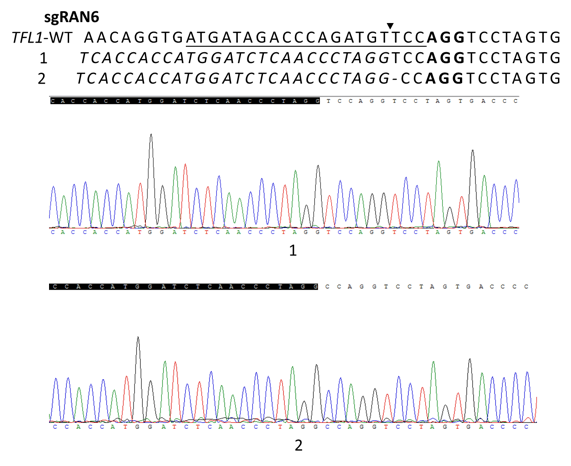
**

**Supplementary Figure 14. DNA sequence of mutant cDNA of *AP1*.**


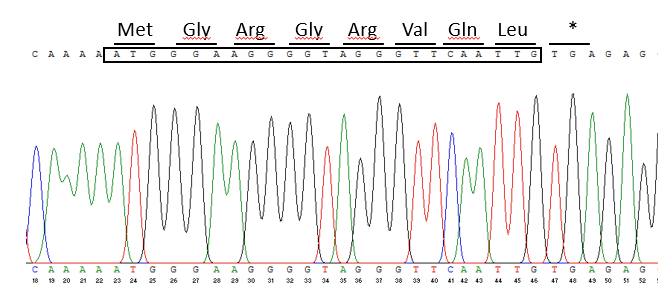


**Supplementary Figure 15. DNA sequence of mutant cDNA of *TFL1*.**


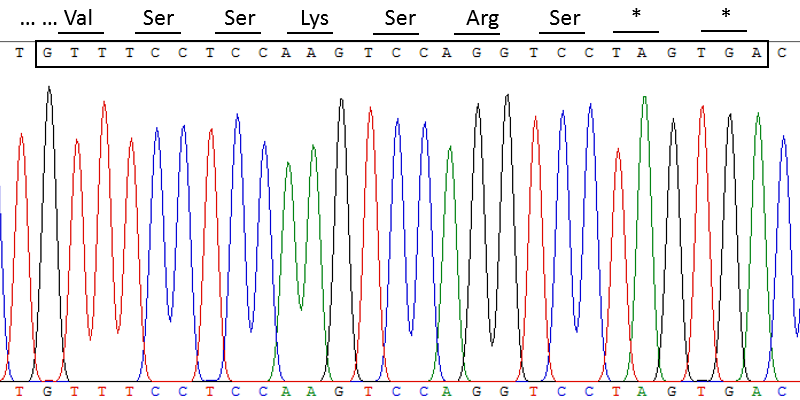

Supplement: Supplementary file 1 — Supplementary information [file 41438_2019_179_MOESM1_ESM.docx]
